# Movements of Wolves at the Northern Extreme of the Species' Range, Including during Four Months of Darkness

**DOI:** 10.1371/journal.pone.0025328

**Published:** 2011-10-04

**Authors:** L. David Mech, H. Dean Cluff

**Affiliations:** 1 Northern Prairie Wildlife Research Center, U.S. Geological Survey, Jamestown, North Dakota, United States of America; 2 Department of Environment and Natural Resources, Government of the Northwest Territories, Yellowknife, Northwest Territories, Canada; University of Pretoria, South Africa

## Abstract

Information about wolf (*Canis lupus*) movements anywhere near the northern extreme of the species' range in the High Arctic (>75°N latitude) are lacking. There, wolves prey primarily on muskoxen (*Ovibos moschatus*) and must survive 4 months of 24 hr/day winter darkness and temperatures reaching −53 C. The extent to which wolves remain active and prey on muskoxen during the dark period are unknown, for the closest area where information is available about winter wolf movements is >2,250 km south. We studied a pack of ≥20 wolves on Ellesmere Island, Nunavut, Canada (80°N latitude) from July 2009 through mid-April 2010 by collaring a lead wolf with a Global Positioning System (GPS)/Argos radio collar. The collar recorded the wolf's precise locations at 6:00 a.m. and 6:00 p.m. daily and transmitted the locations by satellite to our email. Straight-line distances between consecutive 12-hr locations varied between 0 and 76 km. Mean (SE) linear distance between consecutive locations (n = 554) was 11 (0.5) km. Total minimum distance traveled was 5,979 km, and total area covered was 6,640 km^2^, the largest wolf range reported. The wolf and presumably his pack once made a 263-km (straight-line distance) foray to the southeast during 19–28 January 2010, returning 29 January to 1 February at an average of 41 km/day straight-line distances between 12-hr locations. This study produced the first detailed movement information about any large mammal in the High Arctic, and the average movements during the dark period did not differ from those afterwards. Wolf movements during the dark period in the highest latitudes match those of the other seasons and generally those of wolves in lower latitudes, and, at least with the gross movements measurable by our methods, the 4-month period without direct sunlight produced little change in movements.

## Introduction

Animal movements, daily, seasonal, and annual, reveal a great deal of information about a species and its natural history [1], and considerable progress has been made studying such movements. Wolf (*Canis lupus*) movements have been well studied throughout much of the species' circumpolar range [2].

However, information about wolf movements near the northern extreme of the species' range in the High Arctic (>75°N latitude) are lacking, and that area is of interest to study because that is the only region at which wolf movements during winter must take place during 4 months of 24 hr/day darkness and in temperatures as low as −53 C [3]. The closest winter wolf movement study took place >2,250 km south, far south of where it is even dark for 24 hr/day [4]. In addition, no information is available about the movements for any large mammal at 80°N. latitude, so the effect of 24 hr/day darkness on large-mammal movements there is unknown. Furthermore, in some areas at that latitude, wolves prey primarily on muskoxen (*Ovibos moschatus*) [5], [6], [7], and no information is available about wolf movements where the wolf's main prey is muskoxen. This type of information is of intrinsic interest [1] and also as a baseline for gauging the results of climate change, for conditions are changing most rapidly in the Arctic [8].

We studied the movements of a pack of ≥20 wolves at 80°N latitude for 9 months including the 4-month dark period in an area where their main prey was muskoxen.

## Methods

We conducted this study primarily on the Fosheim Peninsula of Ellesmere Island near Eureka (80°N, 86°W), Nunavut, Canada. A weather station is located at Eureka in the east central part of the Fosheim Peninsula north of Slidre Fiord. The amount of daylight in the area varies from 24 hours per day from 16 April to 26 August to 24 hours of darkness per day from 18 October to 23 February. Mean daily temperatures vary from 5.7 C in July to −38.4 C in February. Wolves, muskoxen, and arctic hares (*Lepus arcticus*) have long been common in the area [3], along with a few scattered Peary caribou (*Rangifer tarandus pearyi*), and wolves have denned there for decades or even centuries [9], [10], [11]. Muskoxen are sparsely distributed throughout the area in summer, but the distribution of prey in winter is unknown. During summers from at least 1986 through 1997, a pack of three to seven adult wolves occupied much of the northern half of the Fosheim, preyed on muskoxen and arctic hares, and produced pups almost annually in traditional dens in the area [12]. In 1997 and 2000, however, after snow in mid August abnormally covered the area for the rest of the year, muskox and hare numbers crashed, and wolves disappeared from the immediate area, at least during summer [13]. After a few years of more-normal weather, both prey species began to recover. Wolves reappeared in 2003, then began reproducing in 2004 [14] and continued to reproduce each year through 2010. During the latter period, packs of up to 12 adults were observed during summer.

On 9 July 2009 we used a blowgun and dart with 400 mg Telazol® to anesthetize a dominant male wolf from a distance of 1.5 meters. Induction time was 3 minutes, and the animal appeared fully recovered 3.5 hr later. These methods complied with the guidelines recommended by the American Society of Mammalogists [15] and the requirements of the Nunavut Department of Environment (Wildlife Research Permit WL2009-042). We weighed the wolf at 41 kg, estimated his age at 9 years based on tooth wear [16], ear-tagged him, drew blood from his brachio-cephalic vein, and collared him with a Telonics™ Global-Positioning System (GPS)/Argos™ collar that weighed 960 gm and also contained a very high frequency (VHF) transmitter. (Mention of brand names does not constitute endorsement by the U.S. Government.)

We tracked the collared wolf and his pack for various periods from the ground via his VHF signal from 9 to 16 July 2009. On 15 July, 2009, we homed in on this wolf with a helicopter and observed him and packmates around a den.

**Figure 1:**
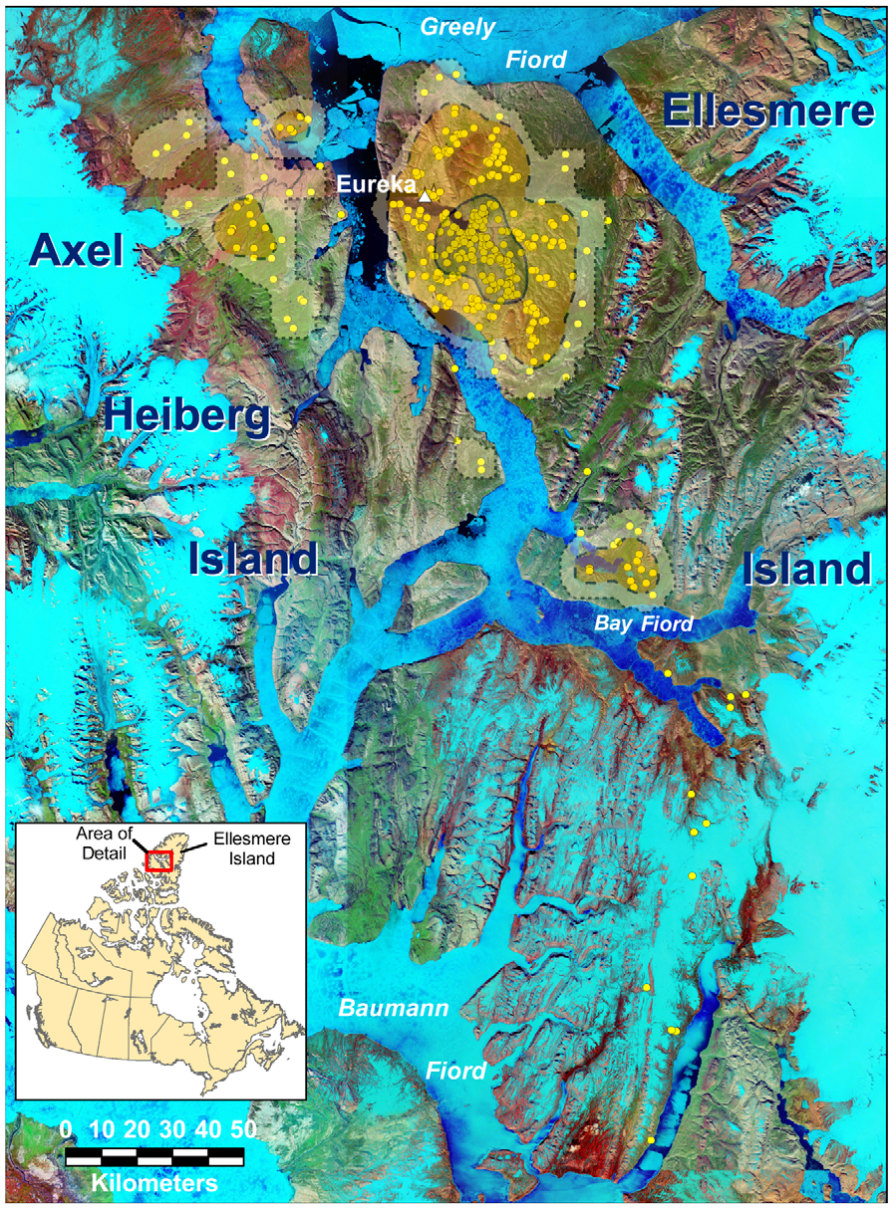
Ellesmere Island, Nunavut, Canada and vicinity with 554 locations of a pack of ≥20 wolves. This pack was monitored by a global-positioning system radio collar 9 July 2009 through 12 April 2010. The dotted line is the 99% probability contour, the dashed line is the 95% contour, and the solid line, the 60% probability.

The GPS collar recorded the collared wolf's locations at 6:00 a.m. and 6:00 p.m. daily from 9 July 2009 through 12 April 2010, and every 4 days it transmitted the locations by Argos satellite to us by email. We plotted the locations on Google Earth™ and in ArcMap™ 9.3.1 (ESRI®, Redlands, CA, USA), and estimated home range with the Animal Movement extension [17] for ArcView 3.3™ (ESRI) based on the fixed-kernel method, using the 99% kernel contours (Fig. 1). We excluded from the home-range calculation 15 locations during a 263-km (straight-line distance) foray out of the main range. Five separate use polygons resulted, and we added their areas to determine total home range area.

The collared wolf visited the vicinity of the Eureka Weather Station 16 times between 7 August 2009 and 16 April 2010, and seven times personnel there counted the wolves, photographed them, and emailed us the photos (Table 1). When the collared wolf stopped moving for several days after 12 April 2010, weather station personnel located his carcass by GPS, retrieved it for us, and we necropsied it. Other researchers in the general area also reported to us observations they made of the wolf pack.

**Table 1:** Observations of a radio-tagged pack of wolves by various workers in the area of Eureka, Ellesmere Island, Nunavut, Canada during 2009–2010.

	No. of Wolves Seen			
Date	Adults	Pups	Observers	Remarks
15 July 09	12	3	Authors	Den
16 July 09	9		Authors	
∼20 July 09	8^a^		Other researchers	Den
7 Aug 09	13		Other researchers	Eureka
12 Sep 09	14	4	Weather Station personnel	Eureka
16 Sep 09	24^b^		Weather Station personnel	Eureka
2 Oct 09	28	4	Other researchers	Eureka
15 Dec. 09	20–25^c^		Weather Station personnel	Eureka
3 Feb. 10	>18^d^		Weather Station personnel	Eureka
4 Mar 10	12	8	Weather Station personnel	Eureka
9 Mar 10	12	8	Weather Station personnel	Eureka
13 Apr 10		9	Weather Station personnel	Eureka
20 Apr 10		6	Weather Station personnel	Eureka

a Based on photo.

b Including “lots of pups”.

c Photos show ≥20.

d Photos show ≥15.

We used program R [18] to examine monthly mean minimum distances traveled via the Dunnett-Tukey-Kramer Pairwise-Multiple-Comparison Test adjusted for unequal variances and unequal sample sizes [19].

## Results

The collared wolf was with a group of four other wolves when we darted him and had just been dominating one of them [20]. Those wolves remained in the immediate vicinity and joined the collared wolf when he recovered, and all traveled 5–10 km away together. Three of the wolves then left the collared wolf, and he followed the adult female for about 15 more km to a den of pups. From a helicopter we observed the collared wolf near a den of ≥3 pups along with ≥11 other adult wolves. Observations of this pack of wolves throughout the study indicated that the pack consisted of a minimum of 20 animals including ≥9 pups (Table 1).

We received all except one of the 555 possible locations recorded by the GPS collar. Straight-line distances between consecutive 12-hr locations varied between 0 and 75.9 km. Mean (SE) linear distance between consecutive locations was 11 km (0.5 km, n = 554), and mean (SE) linear distance between consecutive locations that were >500-m apart was 12 km (0.5 km, n = 482). Total minimum distance traveled was 5,979 km. The collared wolf, and presumably his pack, covered an area of 1,642 km^2^ between 9 July 2009 and 18 October 2009 on the Fosheim Peninsula of Ellesmere Island (Fig. 1). They then crossed some 13 km of a newly frozen fiord just west of Ellesmere Island to Axel Heiberg Island. Between then and 18 February 2010, they crossed to Axel Heiberg and back several times, covering an area there of 65 by 55 km on the east-central lobe of that island. They also later made another round trip to part of Axel Heiberg several km to the southeast in early March. The Axel Heiberg Island locations expanded their range by 1,960 km^2^ (Fig. 1), yielding a total home range of 6,640 km^2^.

Besides the collared pack's usual movements around the Fosheim Peninsula and Axel Heiberg Island, at least the collared animal made a 263-km (straight-line distance) foray to the southeast during 19–29 January 2010, returning from 29 January to 1 February. During the return trip, the animal covered an average of 41 km/day straight-line distances between consecutive 12-hour locations. We have no direct evidence about whether the collared wolf was accompanied by the rest of the pack. However, patterns of consecutive locations later determined to indicate prey carcasses visited (>2 consecutive 12-hr locations the same) were no different on this foray than during movements before and after when the full pack was travelling together. We believe this behavior indicates that the pack accompanied the collared wolf.

**Figure 2:**
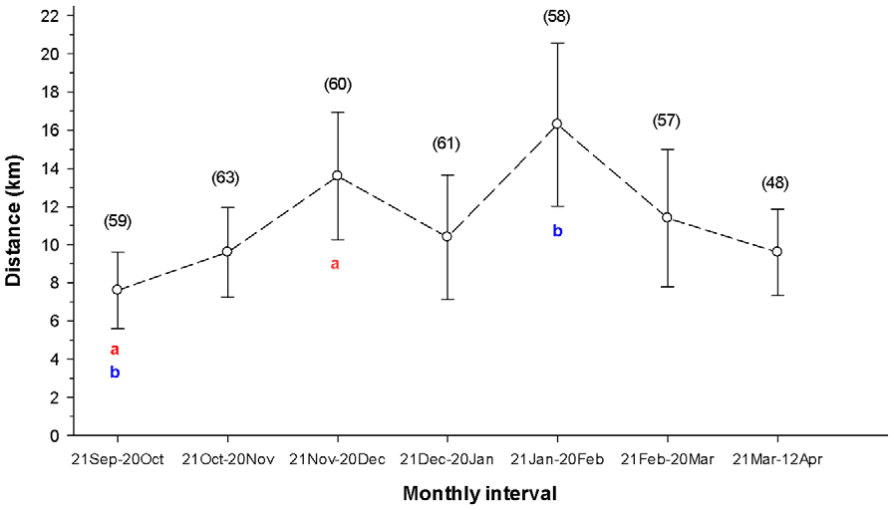
Monthly mean distances between consecutive 12-hr locations of a pack of ≥20 wolves. This pack was monitored by a global-positioning system radio collar on Ellesmere Island, Nunavut, Canada, 9 July 2009 through 12 April 2010. Bars represent 95% confidence intervals. Numbers in parentheses are sample sizes, and like letters indicate significant differences. The mean consecutive distance in September–October is significantly different in pairwise comparisons with those in November–December and January–February.

The pack's mean distances traveled between consecutive 12-hour locations among monthly periods did not differ significantly (*P*≤0.05) except that the mean for September–October was significantly less than those for November–December and January–February (Fig. 2). Such movements from 21 September to 21 October were significantly shorter (8 km) than during the dark period from 21 October to 21 February (12 km), but the average movements during the dark period were not significantly different from those afterwards (10 km). The pack's longest movements between consecutive 12-hr locations increased throughout winter and peaked on 5 March 2010 at 80 km.

The collared wolf died about 12 April 2010. The animal was emaciated and had an enlarged spleen. Some 75% of dogs with enlarged spleens suffer from the cancer hemangiosarcoma, which can cause, among other symptoms, loss of appetite (http://www.dog-health-guide.org/causeforenlargedcaninespleen.html). Our collared wolf would have been an estimated 10-years old at death, uncommonly old for a wild wolf [2].

## Discussion

Our study pack of ≥20 wolves covered an area of 6,640 km^2^ over a period of 8.5 months, several times larger than that of any other known wolf pack [21], except possibly that reported for what appeared to be an itinerant pack in Alaska [22]. No doubt the wide travels of our pack were related to attempts to find and kill vulnerable prey all of which are sparsely distributed in the region. The daily travels of our pack also exceeded those of most other packs [23].

Our study pack traveled throughout winter and the 4-months of 24-hr/day period of darkness at distances similar to those before and after this period. That is, the animals maintained their regular travel schedule throughout the dark period. Although the longest distances between consecutive 12-hr locations increased during the dark period, they continued increasing into early March when the sun was again visible, so this increase was probably unrelated directly to the light regimen. Conceivably the increased longer movements were related to the approaching breeding season which occurs in late March and early April [24].

Our pack's longest 12-hr, straight-line movement of 75.9 km may represent a record for a well-documented distance. There is one undocumented claim of wolves traveling 200 km in a day when hunted [25], and documented records of up to 72-km travel distance (as opposed to a straight-line distance) in 24 hr [23]. For periods ≤2 hr between locations in wooded areas, the straight-line distance can be multiplied by 1.3 to approximate the actual distance traveled [26]. This adjustment would yield a travel distance of 98.7 km for our record, but of course this correction factor would not be completely valid for our 12-hr locations. Furthermore, in our open area, wolves often travel in straighter lines. Nevertheless, it provides some indication of how much longer actual wolf travel distances can be than indicated by straight-line distances between locations.

Our findings extend our knowledge of wolf routine travel capabilities considerably and help explain how wolves can survive as far north as they do, as well as why wolves were originally the most widely distributed non-human terrestrial mammal [27].

Our data also answer the question as to how wolves contend with the dark period in the highest latitudes of the species' range. We conclude that at least with the gross movements measurable by our methods, the 4-month period without direct sunlight produced little change in wolves' movement patterns.
